# The use of lipid-based nanocarriers for targeted pain therapies

**DOI:** 10.3389/fphar.2013.00143

**Published:** 2013-11-21

**Authors:** Susan Hua, Sherry Y. Wu

**Affiliations:** ^1^School of Biomedical Sciences and Pharmacy, The University of NewcastleCallaghan, NSW, Australia; ^2^Department of Gynecologic Oncology, The University of Texas MD Anderson Cancer CenterHouston, TX, USA

**Keywords:** pain, inflammation, liposomes, nanocarriers, targeted drug delivery

## Abstract

Sustained delivery of analgesic agents at target sites remains a critical issue for effective pain management. The use of nanocarriers has been reported to facilitate effective delivery of these agents to target sites while minimizing systemic toxicity. These include the use of biodegradable liposomal or polymeric carriers. Of these, liposomes present as an attractive delivery system due to their flexible physicochemical properties which allow easy manipulation in order to address different delivery considerations. Their favorable toxicity profiles and ease of large scale production also make their clinical use feasible. In this review, we will discuss the concept of using liposomes as a drug delivery carrier, their *in vitro* characteristics as well as *in vivo* behavior. Current advances in the targeted liposomal delivery of analgesic agents and their impacts on the field of pain management will be presented.

## INTRODUCTION

Targeted drug delivery provides effective, precise, and safe therapeutic interventions for treatment of diverse disease conditions, by limiting toxic side effects and/or increasing drug action. Effective drug targeting depends on several factors that are either carrier or target related. The drug carrier must be stable, protect the drug from degradation, protect the body from harmful side effects, and allow delivery to the target cell population *in vivo* (Koning et al., 2002). The target must be well accessible for the drug-targeting system and must display specific cell-surface molecules that allow selective targeting and efficient drug delivery (Vingerhoeds et al., 1994; Willis and Forssen, 1998; Ding et al., 2006). The field of site-specific drug delivery has been continuously explored to develop formulations with a therapeutically acceptable degree of target specificity. Many different approaches using various physical and biochemical principles have been proposed and examined, with targeted liposomes as a carrier for both hydrophobic and hydrophilic drugs having attracted much attention.

## LIPOSOMES AS DRUG DELIVERY CARRIERS

Liposomes have long been considered good candidates for efficient drug carrier and delivery systems. They have been used as delivery vehicles for stabilizing drugs, overcoming barriers to cellular and tissue uptake, and for directing their contents toward specific sites *in vivo* (Senior, 1987; Oku and Namba, 1994; Vingerhoeds et al., 1994; Woodle et al., 1994; Torchilin, 1996; Willis and Forssen, 1998; Bendas, 2001; Maruyama, 2002; Moghimi and Szebeni, 2003; Metselaar and Storm, 2005; Ding et al., 2006). The unique ability of liposomes to entrap drugs both in an aqueous and a lipid phase make such delivery systems attractive for hydrophilic and hydrophobic drugs. Hydrophobic molecules are intercalated within the bilayer membrane, and hydrophilic molecules can be entrapped in the internal aqueous region. Additionally, by virtue of their large aqueous interior and biocompatible lipid exterior, they offer a possible means of local delivery of a large variety of drug structures, from small molecules to macromolecules such as proteins and DNA, to the site of interest while reducing systemic toxicity (Senior, 1987; Oku and Namba, 1994; Torchilin, 1996; Ulrich, 2002; Sahoo and Labhasetwar, 2003; Ding et al., 2006).

Liposomes offer several advantages over other delivery systems. Liposomes are generally considered non-toxic, biodegradable, and non-immunogenic, as they are typically composed of naturally occurring lipids. Association of a drug with liposomes generally prolongs circulation half-life, reduces volume of distribution, and lowers systemic toxicity. Moreover, the drug is protected from early degradation, inactivation, and dilution in circulation (Oku and Namba, 1994; Torchilin, 1996; Laverman et al., 1999; Ulrich, 2002; Sahoo and Labhasetwar, 2003). *In vivo* behavior of liposomes can be easily modified by changing their characteristics, such as size, lipid composition, and charge (Senior, 1987; Oku and Namba, 1994; Willis and Forssen, 1998; Laverman et al., 1999; Ulrich, 2002). In addition, the liposome surface can be modified with polymer structures such as poly(ethylene glycol) (PEG), which inhibits macrophage uptake and thereby increases liposome circulation time, and with targeting moieties such as antibodies or peptides (Senior, 1987; Oku and Namba, 1994; Torchilin, 1994; Woodle et al., 1994; Maruyama, 2002; Moghimi and Szebeni, 2003). Site-directing ligands incorporated into the liposome membrane surface therefore have been investigated intensely in an effort to further enhance the selectivity of liposomal drug delivery (Sawant and Torchilin, 2012; Allen and Cullis, 2013; Koshkaryev et al., 2013). Unlike solid polymeric carrier systems, liposome membranes are dynamic structures, allowing surface-coupled ligands a greater degree of freedom with the ability to move about within the bilayer plane, positioning themselves for optimal substrate interactions (Willis and Forssen, 1998). Critical factors for successful *in vivo* delivery of ligand-targeted liposomes will involve selection of accessible and appropriate targets, use of ligands with adequate selectivity and affinity for these targets, and suitable liposome surface coupling methods for correct presentation of ligands to their binding sites (Vingerhoeds et al., 1994; Torchilin, 1996; Willis and Forssen, 1998; Metselaar and Storm, 2005; Ding et al., 2006). The benefit of liposomes as therapeutic carriers stimulates the accumulation of novel experiences in the practical aspects of liposomes, as well as new developments in basic research.

## *IN VIVO* STABILITY, BIODISTRIBUTION, AND BIOAVAILABILITY OF LIPOSOMES

Several major hurdles must be overcome in order to prolong liposome circulation times. These include stabilizing the vesicles against leakage of entrapped contents, avoiding opsonization, and minimizing removal by the reticuloendothelial system (RES; Willis and Forssen, 1998). The rate at which liposomes are cleared depends on their size, surface charge, and stability (Oku and Namba, 1994; Laverman et al., 1999; Ishida et al., 2001; Ulrich, 2002). The presence of a high electrostatic surface charge promotes the interaction of liposomes with biomolecules that could serve as opsonins and with cells (Laverman et al., 1999; Ishida et al., 2001). In general, unmodified large liposomes are cleared more rapidly than small, neutral, or positively charged liposomes (Oku and Namba, 1994; Laverman et al., 1999; Ishida et al., 2001; Ulrich, 2002). Previous studies have demonstrated that the liver removes large, charged liposomes rapidly, with spleen clearance half-life of less than 1 h (Chrai et al., 2002). The presence of cholesterol is another important factor both for enhancing stability against leakage and in minimizing phospholipid exchange (Willis and Forssen, 1998; Laverman et al., 1999). This minimizes lipid exchange with other structures in the circulation (red blood cells, lipoproteins), which can lead to depletion of the high phase transition temperature lipids and their replacement with less physiologically stable components (Willis and Forssen, 1998; Laverman et al., 1999; Ulrich, 2002).

A major concern in using liposomes for therapeutic purposes is their fast removal from blood circulation by components of the RES. The RES is the major site of liposome accumulation after systemic administration. Primary organs associated with the RES are the liver, spleen, kidneys, lungs, bone marrow, and lymph nodes (Senior, 1987; Oku and Namba, 1994; Vingerhoeds et al., 1994; Ishida et al., 2001; Chrai et al., 2002). The liver exhibits the largest capacity for uptake, whereas the spleen can accumulate liposomes so that its tissue concentration is 10-fold higher than those of other organs (Chrai et al., 2002). Removal of liposomes from the blood is attributed to phagocytic cells that reside in the RES and is mediated through direct interactions between those cells and the liposomes (Senior, 1987; Oku and Namba, 1994; Vingerhoeds et al., 1994; Ishida et al., 2001; Chrai et al., 2002). Although clearance of liposomes by the RES occurs predominantly after opsonization of the vesicles, that is the adsorption of plasma proteins (e.g. immunoglobulins, fibronectin, complement components, C-reactive protein) onto their surface, *in vitro* studies have shown that liposomal uptake into macrophages can also occur in the absence of serum proteins (Ishida et al., 2001; Chrai et al., 2002). The extent of opsonization decreases with a decrease in liposome size from 800 to 200 nm in diameter (Chrai et al., 2002). Small liposomes could not support opsonic activity, whereas the larger ones did so substantially. The profound effect of size on complement recognition affects liver uptake, depending on the extent of liposome opsonization (Laverman et al., 1999; Ishida et al., 2001; Chrai et al., 2002). One of the major steps in improving circulation time and preventing removal by RES was sterically stabilizing the liposomes through the introduction of PEG modification (Oku and Namba, 1994; Torchilin, 1994, 1996; Vingerhoeds et al., 1994; Woodle et al., 1994; Willis and Forssen, 1998; Maruyama, 2002; Ulrich, 2002; Moghimi and Szebeni, 2003). More specifically, stabilization of liposomes with PEG creates a local surface concentration of highly hydrated groups which sterically inhibits both electrostatic and hydrophobic interactions with a variety of serum proteins or cells, thus resulting in a reduced uptake by cells of the RES (Ishida et al., 2001). Many targeting systems with promising outlook based on *in vitro* results have faced the above problems when tested *in vivo *(Sahoo and Labhasetwar, 2003). Therefore, having an understanding of the events that take place *in vivo* is essential for the design of particles with optimal circulation profiles.

The accumulation of liposomes at the target site is a prerequisite but does not necessarily guarantee a therapeutic effect of the encapsulated drug. Therefore, the crucial role of the liposome-cell interaction has to be taken into account (Vingerhoeds et al., 1994; Willis and Forssen, 1998; Ulrich, 2002). Multiple factors such as activation state of the target cell or size, charge, sterical stabilization, and pH-dependence of the liposomes have an important impact on this interaction (Vingerhoeds et al., 1994; Willis and Forssen, 1998; Laverman et al., 1999; Ulrich, 2002; Muro and Muzykantov, 2005). The cellular incorporation of liposomal content can occur in different ways: (i) extracellular release of the soluble content and uptake via diffusion or pore formation; (ii) liposomal fusion within the cell membrane followed by an intracellular release of the liposomal content; and (iii) active uptake of the liposomes via an endocytotic or phagocytotic pathway (Vingerhoeds et al., 1994; Willis and Forssen, 1998; Bendas, 2001; Ulrich, 2002). In receptor-mediated endocytosis, small particles (<150 nm diameter) bind to cell surface receptors and are taken up by clathrin-coated pits to form coated vesicles. After internalization, the clathrin coat is removed and the vesicle fuses with lysosomes, which induces the breakdown of the lipids and release of their contents. Large particles (>150 nm), on the other hand, are taken up principally by phagocytosis, which is usually limited to specific cells such as macrophages but can be induced in many other cell types with appropriate ligands. In both cases, liposomes could either be degraded in the low pH environment, or they could fuse directly with the endosomal or lysosomal membrane (Willis and Forssen, 1998; Ulrich, 2002). In addition, macromolecules can cross the endothelial barrier in three ways: (Koning et al., 2002) between the cells, through cell junctions (paracellular); (Ding et al., 2006) through the endothelial cell, via pores; and (Vingerhoeds et al., 1994) transcellularly, via shuttling vesicles and specific receptors (van Hinsbergh, 1997; Antohe et al., 2004). It is generally believed that the charge and compactness of the endothelial matrix contribute additionally to the selectivity of the endothelial barrier toward molecules of different size and charge (van Hinsbergh, 1997).

## LIPOSOMES – THERAPEUTIC OPPORTUNITIES

The use of liposomes as drug sustained release systems or as drug delivery systems for passive targeting is well established, with several drug formulations in the clinic or in late clinical trials (Sawant and Torchilin, 2012; Allen and Cullis, 2013; Koshkaryev et al., 2013). Several laboratories have reported the use of liposomes as drug carriers in the treatment of cancer, fungal diseases, and inflammatory or immune diseases (Oku and Namba, 1994; Vingerhoeds et al., 1994; Woodle et al., 1994; Willis and Forssen, 1998; Sahoo and Labhasetwar, 2003; Metselaar and Storm, 2005). Innovative research in liposomal drugs has led to commercialization of several liposomal formulations, including anticancer therapeutics (Doxil^®^ and Myocet^®^) and an antifungal drug formulation (AmBisome^®^). These products have demonstrated improved therapeutic indices over their corresponding conventional drugs by avoiding sensitive tissues and/or increasing delivery to specific targets *in vivo* (Oku and Namba, 1994; Vingerhoeds et al., 1994; Willis and Forssen, 1998). Liposomes offer several advantages over other delivery systems including biocompatibility, capacity for self-assembly, ability to carry large payloads of active agent, and a wide range of physical properties that can be modified to control their biological properties (Senior, 1987; Woodle et al., 1994; Torchilin, 1996; Willis and Forssen, 1998; Bendas, 2001; Moghimi and Szebeni, 2003). Additionally, the delivery system itself is pharmacologically inactive with minimal toxicity, and is readily metabolized and cleared from the circulation once its carrier function has been completed (Willis and Forssen, 1998). An advantage that liposomes possess over solid particulate delivery systems is their ability to transport and deliver biologically active molecules without the need for covalent coupling (Willis and Forssen, 1998). To improve upon these therapies, clinically active liposomal delivery systems may need to include site-directed surface ligands to further enhance their selective delivery. The concept of drug targeting and controlled drug delivery is used in attempts to improve the therapeutic index of drugs by increasing their localization to specific organs, tissues, or cells and by decreasing their activity and potential toxic side effects in normal organs (e.g. heart, liver, or kidneys). This concept is especially important for drugs with a narrow therapeutic window which has the potential of having detrimental effects (Vingerhoeds et al., 1994; Willis and Forssen, 1998; Bendas, 2001; Maruyama, 2002).

## USE OF NANOCARRIERS FOR PAIN THERAPIES

**FIGURE 1:**
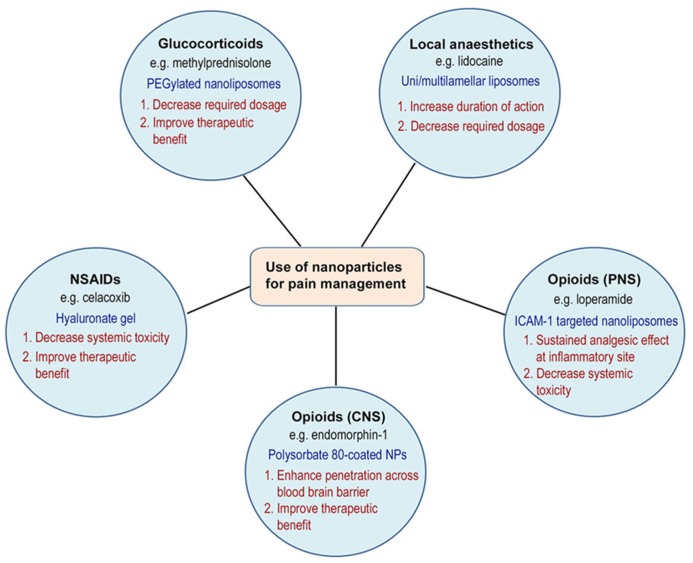
**Use of nanocarriers for pain management**.

Drug delivery systems have been used in pain therapies to improve toxicity or side effect profiles by targeted delivery to specific sites in the body, increase drug upload or bioavailability, and to provide prolonged drug release. For example, an area of interest has been the delivery of opioid-based compounds to target peripheral opioid receptors within injured tissue to promote analgesic and anti-inflammatory activity (Hua and Cabot, 2010). It is well-established that many conventional opioid agonists have been shown to produce potent opioid receptor mediated analgesia when administered locally into injured tissue of rodents, non-human primates, and humans (Stein et al., 2001, 2003; Rittner and Stein, 2005; Rittner et al., 2005). However, with increased blood flow secondary to inflammation, drugs may still be absorbed into the systemic circulation, leading to side effects mediated by activation of central or peripheral opioid receptor activity (e.g., sedation, respiratory depression, dependence, tolerance, nausea, or constipation) (Stein et al., 2001, 2003; Menendez et al., 2005; Rittner et al., 2005; Rittner and Stein, 2005; Sevostianova et al., 2005). This area of research of applying targeted drug delivery and the use of nanocarriers in the management of pain is a novel and exciting area of research, with much potential for growth and clinical benefits. The remainder of the review will focus on the progress made in this area of research in experimental and clinical studies (**Figure 1**).

## EXPERIMENTAL USE OF NANOCARRIERS FOR PAIN THERAPIES

Nanosystems used for delivering compounds intended for pain therapies, such as local anesthetics (de Paula et al., 2012) or non-steroidal anti-inflammatory drugs (NSAIDS), have been reviewed previously (Puglia et al., 2013). The encapsulation of local anesthetics into liposomes, for instance, presents advantages such as slow release, prolonged duration of action, reduced plasma concentrations, and low toxicity to the central nervous and cardiovascular systems. A number of pre-clinical studies have been conducted encapsulating local anesthetics, such as bupivacaine or lidocaine, into multilamellar or unilamellar liposomes using different phospholipid and pH combinations (de Paula et al., 2012). These studies report increased duration of anesthesia and sensory blockade following parenteral administration of these formulations.

Targeted delivery of glucocorticosteroids has been widely studied for the treatment of rheumatoid arthritis and other inflammatory joint conditions (Metselaar et al., 2003, 2004; Metselaar and Storm, 2005). Although corticosteroids are not classified as an analgesic, the pain relieving effects are secondary to their anti-inflammatory activity. Long-circulating PEGylated liposomes containing methylprednisolone or betamethasone have been used to treat Lewis rats with adjuvant-induced arthritis (AIA) both at early (before clinical signs appear) and late (at the peak of the disease) stages of the disease (Avnir et al., 2008). In addition, Ulmansky et al. (2012) showed that intravenous treatment with sterically stabilized nano-liposomes (NSSL) encapsulated with methylprednisolone or betamethasone significantly decreased the severity of adjuvant arthritis in Lewis rats throughout all disease stages. They reported that both subcutaneous and intravenous administration of glucocorticoid-encapsulated NSSL was able to suppress arthritis significantly compared to higher doses of the free drugs or to TNF-α antagonists (Ulmansky et al., 2012).

Non-steroidal anti-inflammatory drugs have long been used as an analgesic and anti-inflammatory agent. However, they are associated with numerous interactions with other medications and have serious side effects to the gastrointestinal tract, kidneys, and cardiovascular system (Rittner et al., 2005; Warner and Mitchell, 2008). Nanocarriers have been used to enhance the efficacy and reduce the toxicity of NSAIDs by targeted delivery to the site of inflammatory pain. A number of topical and parenteral nano-formulations have been utilized and have shown success in preclinical studies (Bansal et al., 2007; Raffin et al., 2012; Tarţău et al., 2012; Dong et al., 2013; Puglia et al., 2013). Dong et al. (2013) recently demonstrated that celecoxib-loaded liposomes embedded into hyaluronate gel was more effective than either single agent in pain control and cartilage protection in a rabbit knee osteoarthritis model following intra-articular injection.

Targeted nanoparticles have recently been engineered to deliver opioids, in particular loperamide HCl, specifically to peripheral opioid receptors to induce analgesic and anti-inflammatory actions for use in painful inflammatory conditions (Hua and Cabot, 2013). Loperamide is a peripherally-selective mu-opioid receptor agonist that does not have analgesic effects following intravenous or oral application due to its physicochemical properties. These nanoparticles are conjugated with antibodies targeted against intercellular adhesion molecule-1 (anti-ICAM-1) which mimics the properties of opioid-containing immune cells. These targeted nanoparticles produced highly significant analgesic and anti-inflammatory effects over the 48-h time course studied following intravenous administration in rats with Complete Freund’s Adjuvant-induced inflammation of the paw. Biodistribution data demonstrated specific localization of the targeted nanoparticles to peripheral inflammatory tissue with no significant uptake into the brain (Hua and Cabot, 2013). Other sustained release systems have also been engineered to prolong the duration of action of opioid analgesics (Ward et al., 2013).

A number of non-lipid-based nanocarrier formulations have also been studied to improve the oral (Martín-Banderas et al., 2012; Tang et al., 2012), intranasal (Kumar et al., 2013; Patel et al., 2013), and CNS delivery of analgesic agents (Liu et al., 2006; Tosi et al., 2007; Chen et al., 2013). Local or systemic administration of endogenous opioid peptides (e.g. β-endorphin) is not viable due to its short half-life in the blood and within inflamed tissue. Liu et al. (2006) demonstrated that opioid peptides, in particular endomorphin-1, adsorbed onto the surface of butylcyanoacrylate nanoparticles and coated with polysorbate 80 could penetrate the blood-brain barrier following intravenous administration to cause analgesia. Tosi et al. (2007) investigated the *in vivo* antinociceptive efficacy of peptide-derivatised nanoparticles loaded with loperamide HCl for delivery to central opioid receptors, and reported a peak percentage of maximum possible effect (% MPE) of 60% at 4 h and a significant sustained release effect for 6 h after tail vein injection of a dose equivalent of 0.7 mg of loperamide HCl in Wistar rats. In addition, Chen et al. (2013) showed that nanoparticles consisting of loperamide and PLGA-PEG-PLGA triblock copolymer coated with poloxamer 188 or polysorbate 80 had improved penetration across the blood-brain barrier in comparison to PLGA-PEG-PLGA nanoparticles and PLGA nanoparticles. These studies demonstrate that the use of surface modification for nanoparticles is an efficient strategy to deliver opioid analgesics to specific sites in the body.

## CLINICAL USE OF NANOCARRIERS FOR PAIN THERAPIES

Although liposomes and nanoparticles present an exciting opportunity to improve the management of a variety of painful conditions, current clinical use is limited and few products appear to be in use for human clinical trials. Liposome encapsulation of local anesthetics, NSAIDs, and opioids has been studied in humans with promising results. For example, liposomal formulations of local anesthetics have been demonstrated to provide significantly prolonged pain relief after surgical procedures and in chronic cancer (de Paula et al., 2012). Gorfine et al. (2011) compared the magnitude and duration of postoperative analgesia from a single dose of bupivacaine extended-release injection with placebo administered intraoperatively via wound infiltration in 184 patients undergoing hemorrhoidectomy in a multicenter, randomized, double-blind, placebo-controlled trial. The results showed that the liposomal formulation significantly reduced pain over 72 h and decreased opioid requirements, compared to placebo (Gorfine et al., 2011). Similarly, Lafont et al. (1996) reported prolonged pain relief in a patient with chronic cancer that lasted for 11 h after injection of a liposomal bupivacaine formulation, compared to 4 h for plain bupivacaine.

The efficacy of topical liposomal NSAID-based formulations has also been demonstrated in clinical studies (Puglia et al., 2013). For example, indomethacin-loaded liposomes incorporated into hydrogels were studied in UVB-induced erythema on healthy human volunteers. The results provided a more prolonged anti-inflammatory effect in comparison to a gel formulation containing free drug, allowing a sustained release of the drug to deeper skin layers (Puglia et al., 2004).

Strategies to restrict the access of opioid agonists to the CNS have also been of major interest in pain research (Menendez et al., 2005; Sevostianova et al., 2005). With regards to incorporation of hydrophilic opioids into liposomal formulations, an extended-release morphine preparation based on a multivesicular lipid suspension foam technology is available in the United States (Rose et al., 2005; Viscusi et al., 2005). This preparation is indicated for pain relief after major surgery (e.g., orthopedic surgery involving lower extremities, lower abdominal surgery, or cesarean delivery) as a single lumbar epidural injection. Studies have demonstrated effective, dose-related analgesia for up to 48 h after a single dose (Rose et al., 2005; Viscusi et al., 2005). Although the safety profile was largely consistent with those for other epidurally administered opioid analgesics, systemic adverse effects were still reported. In fact, the rate of respiratory depression was higher in the liposomal morphine group compared with the intravenous patient controlled analgesia (PCA) fentanyl group, which suggest that patient characteristics are important in choosing an appropriate dose of liposomal morphine (Viscusi et al., 2005). While benefits were seen with its use following cesarean section (Carvalho et al., 2007), another study showed no benefit over traditional opioids following abdominal surgery with breakthrough pain relief still required and a similar side effect profile to traditional opioids (Gambling et al., 2005). To date, the clinical studies for pain therapies have only investigated the use of conventional liposomes which permits passive targeting. It is anticipated that the use of ligand-targeted nanocarriers (active targeting) for pain therapies will further improve the efficacy and side effect profile of the conventional liposome formulations.

## CONCLUSION

This phenomenon of disease-site targeting is believed to play a major role in the enhanced efficacy observed for a variety of drugs when formulated inside lipid vesicles. Despite the clinical need, the use of nano-based therapeutics to target and treat inflammation and pain is only beginning to be exploited. The use of drug-loaded liposomes for this application would be promising for a multitude of acute and chronic pain conditions (e.g., post-operative pain, visceral cancer pain, rheumatoid arthritis, or neuropathic pain). Their use will ultimately lead to improved efficacy, increased duration of action, and improved side effect profile of analgesic and anti-inflammatory therapeutics.

## Conflict of Interest Statement

The authors declare that the research was conducted in the absence of any commercial or financial relationships that could be construed as a potential conflict of interest.
